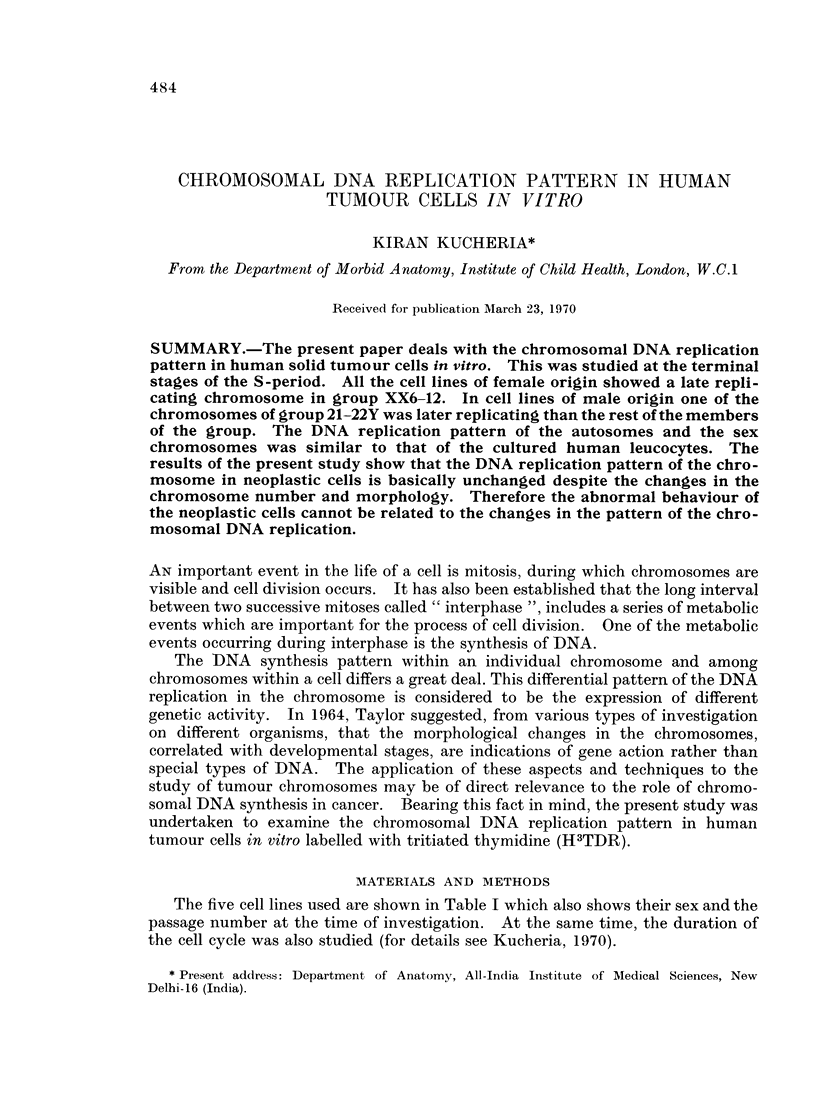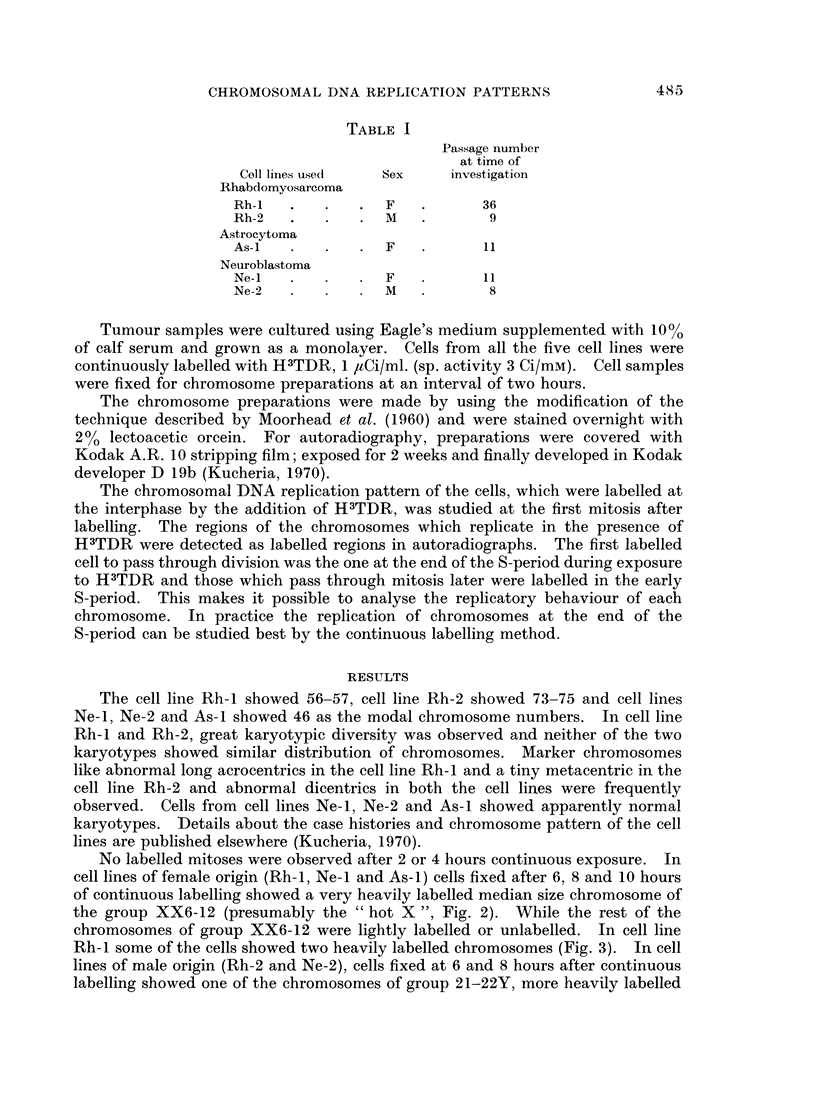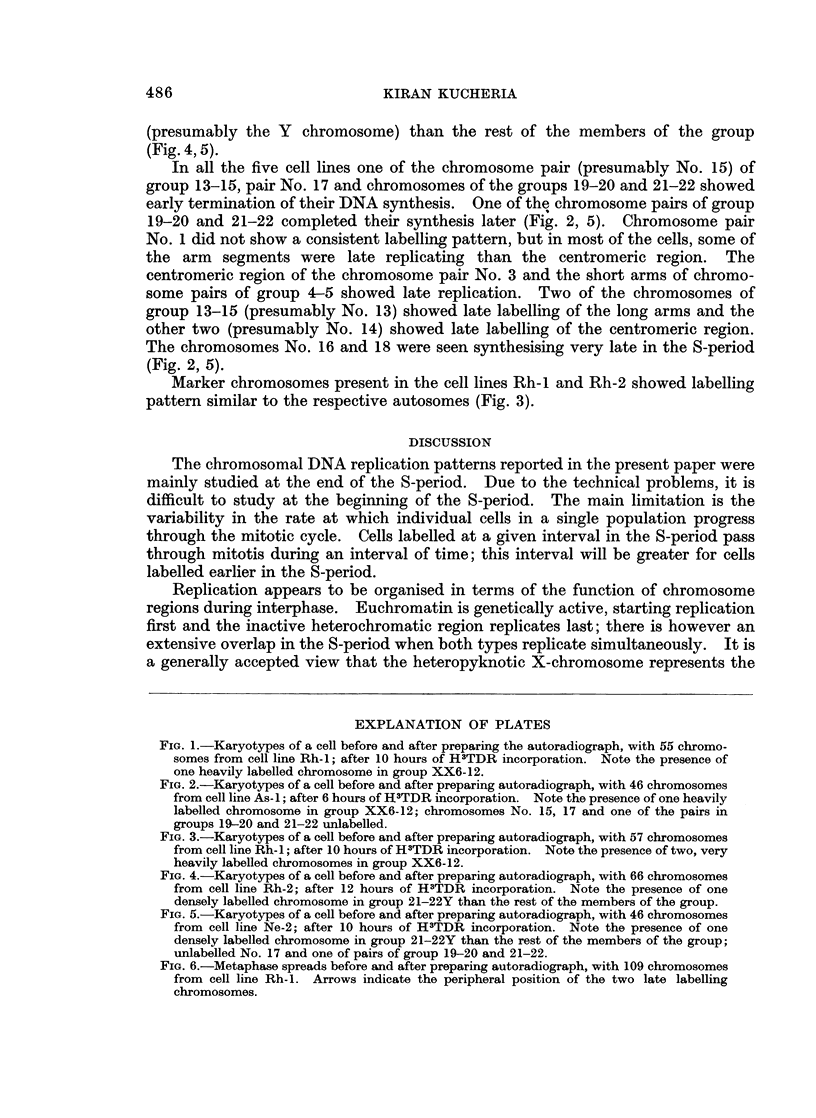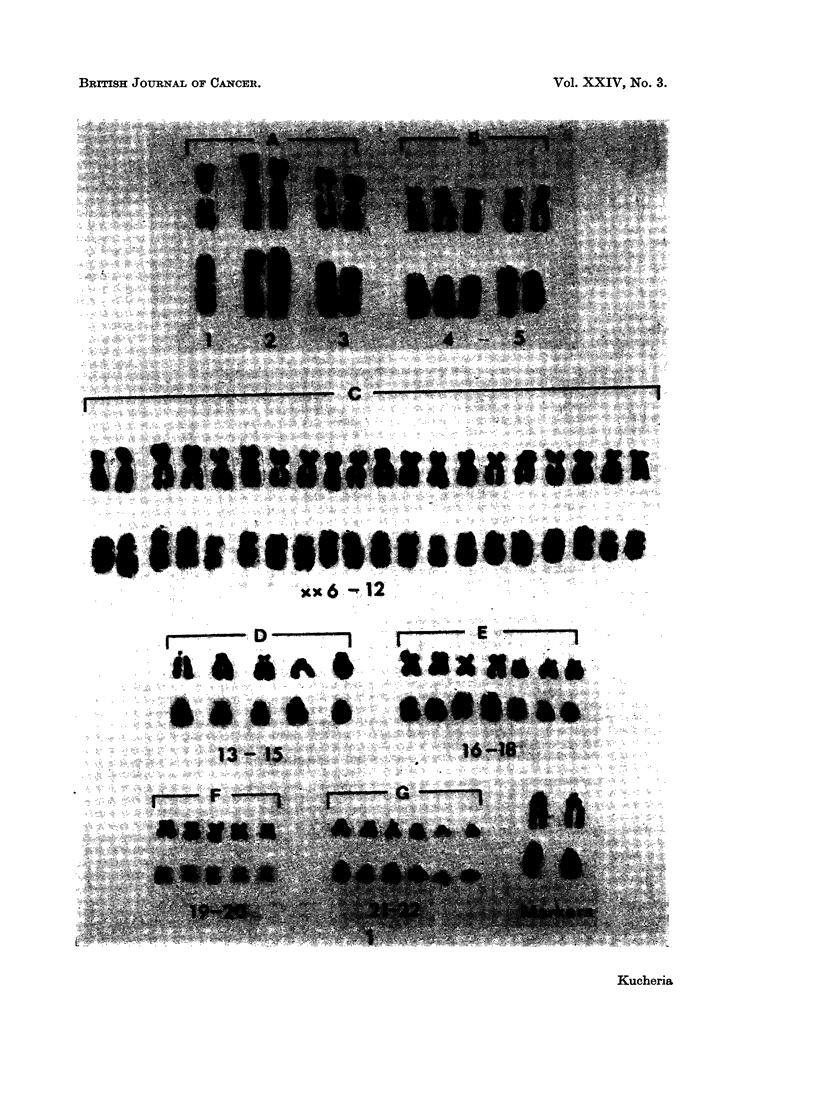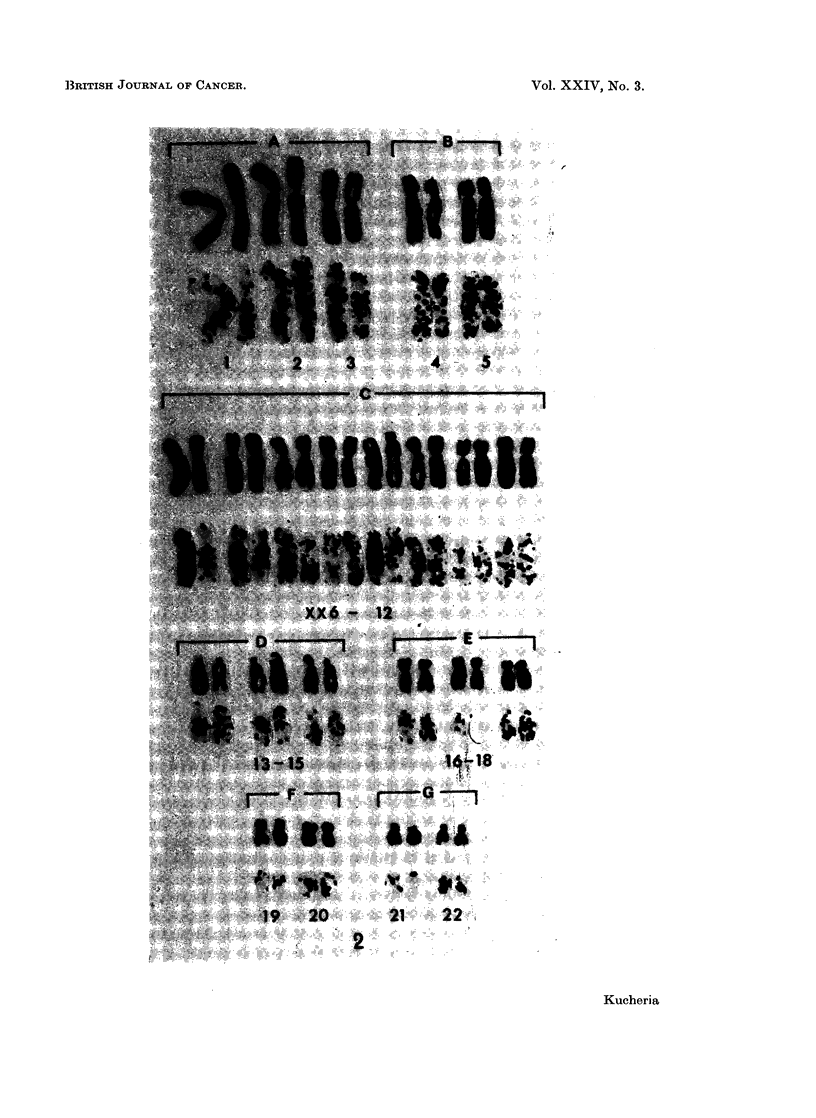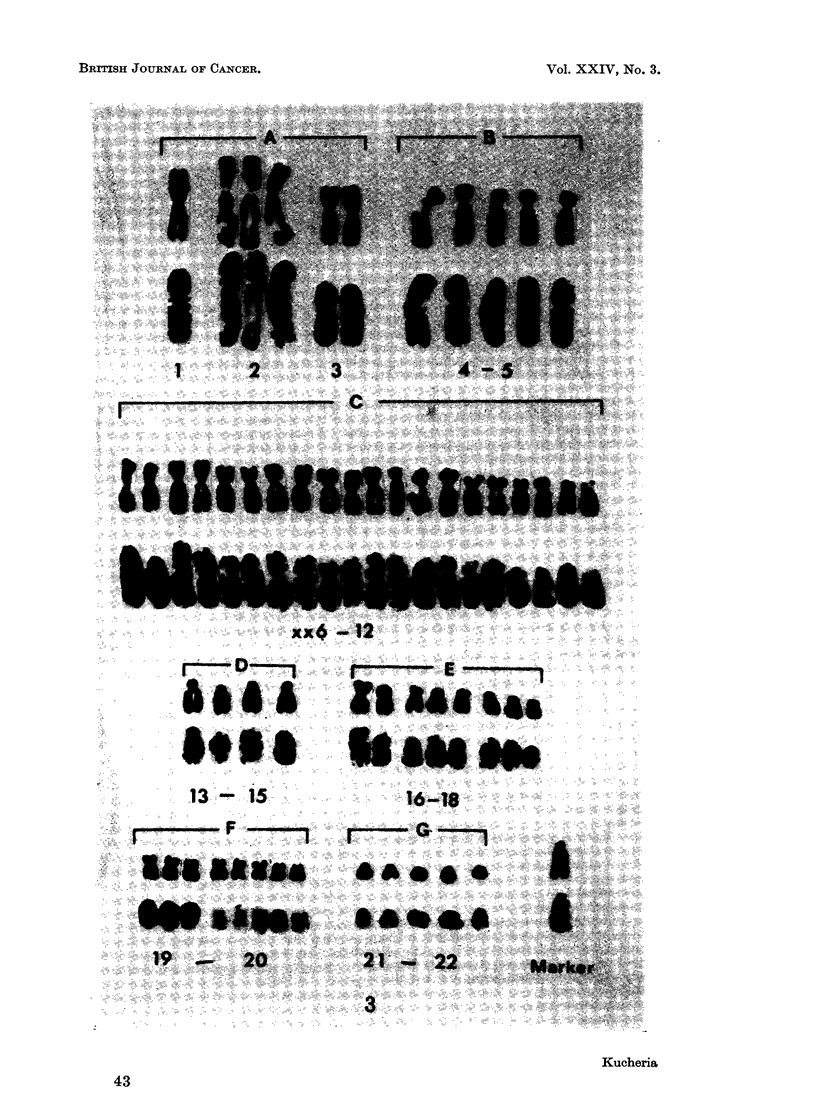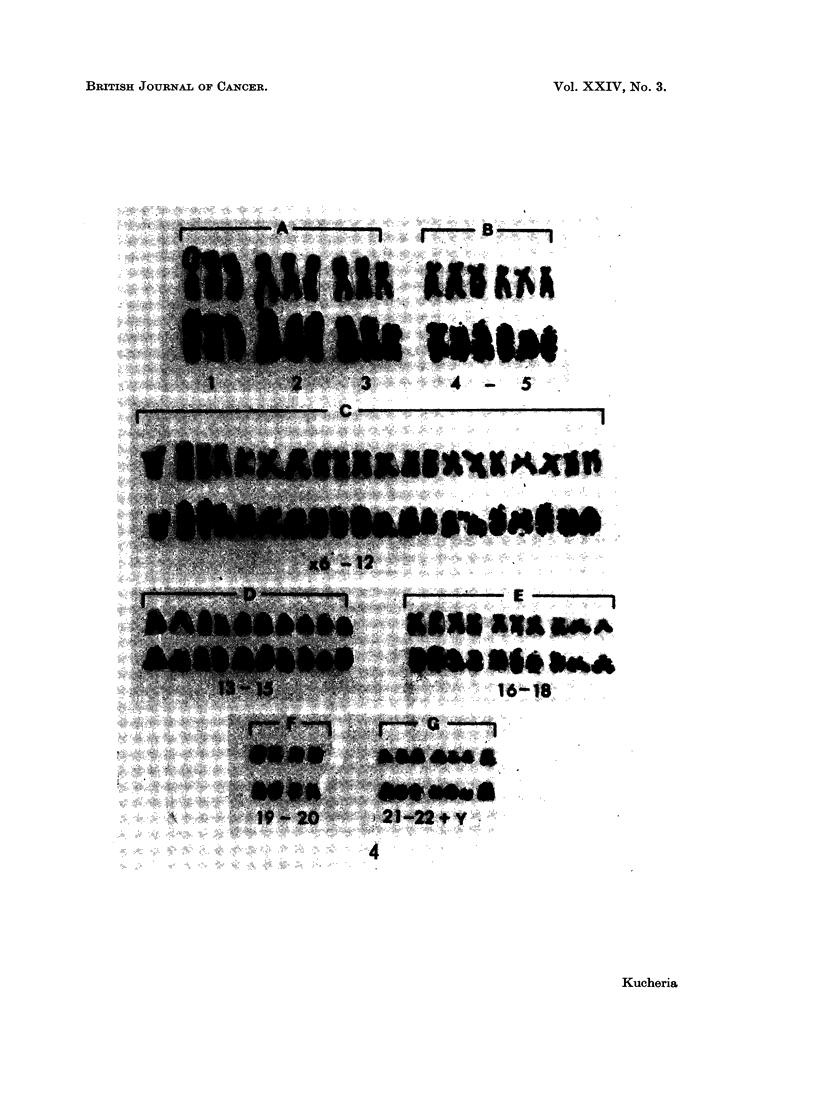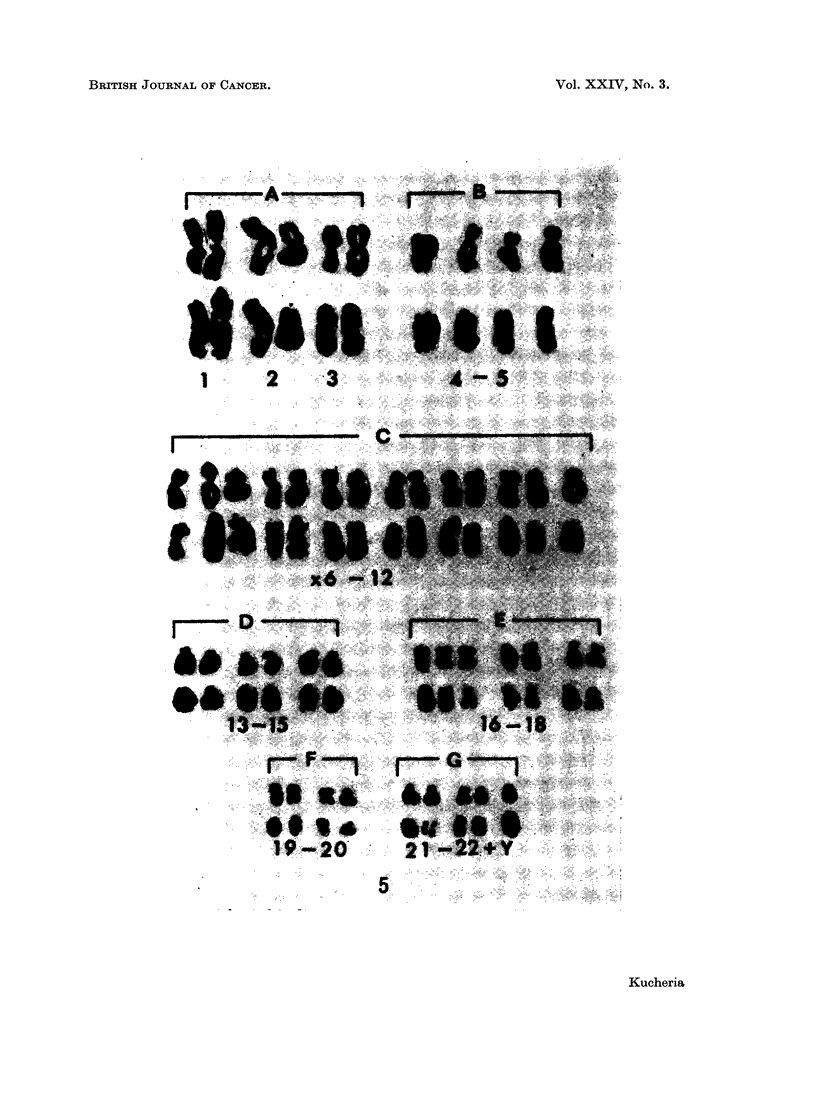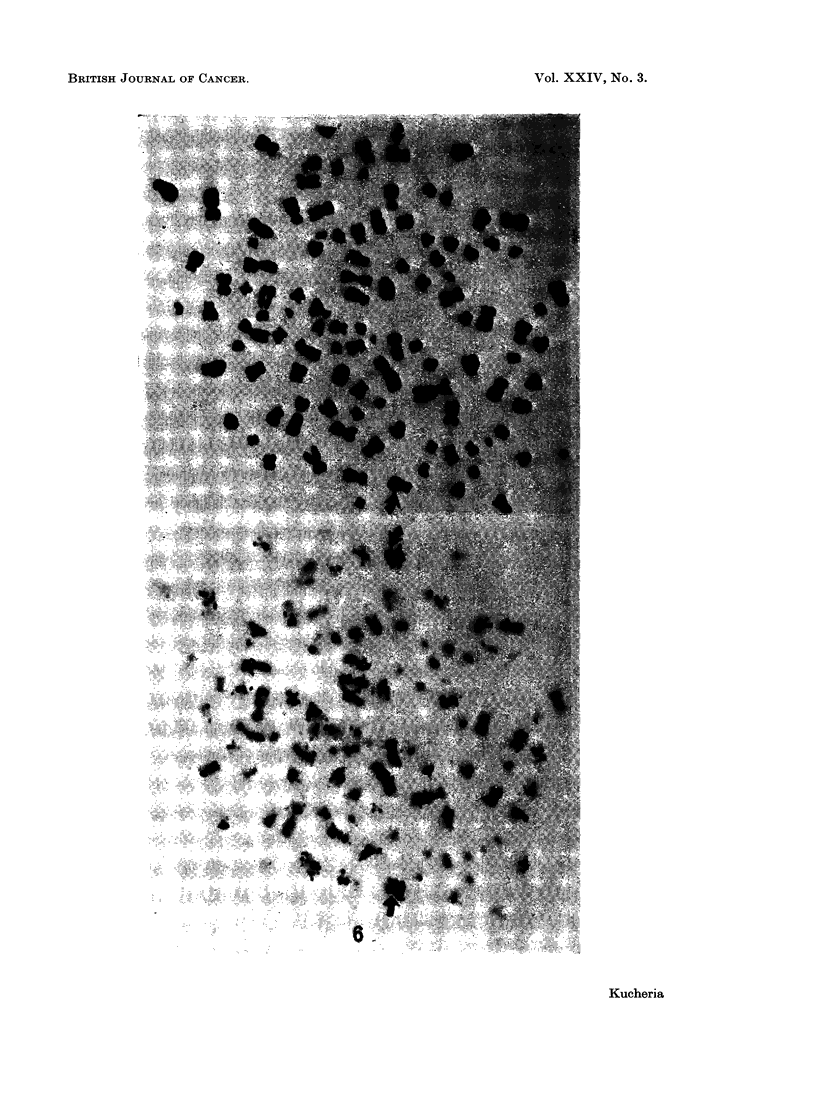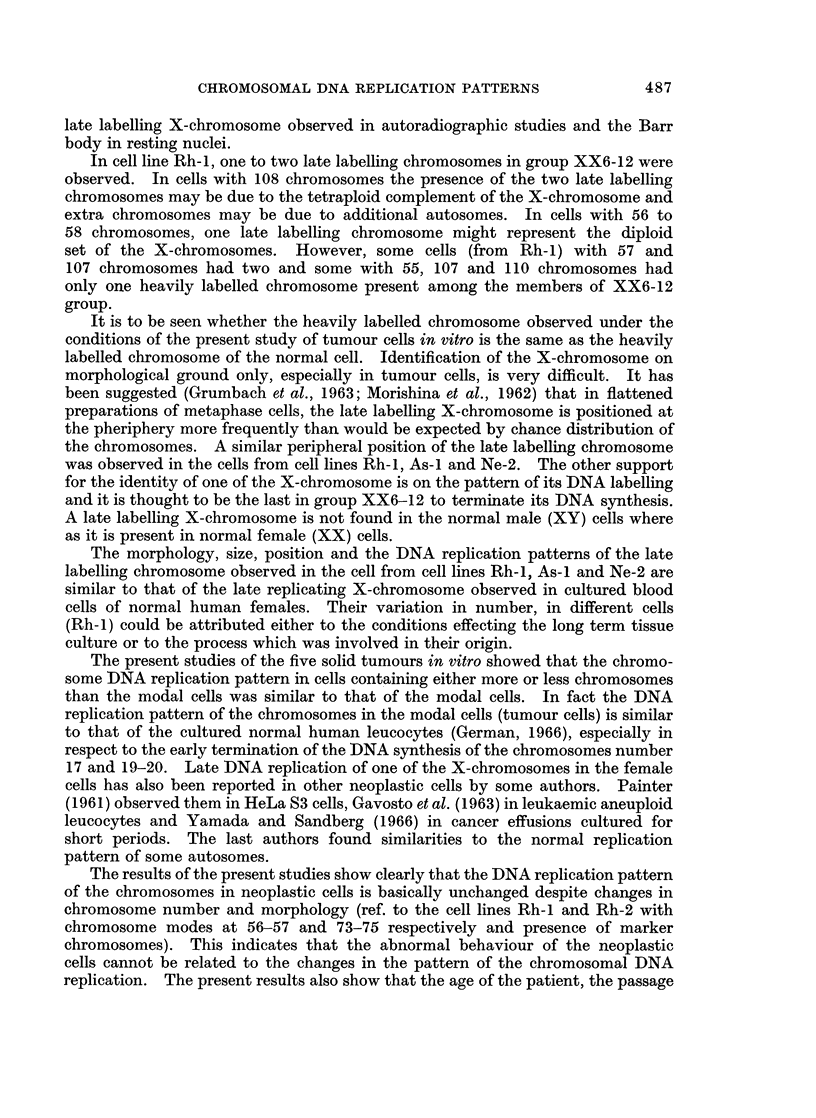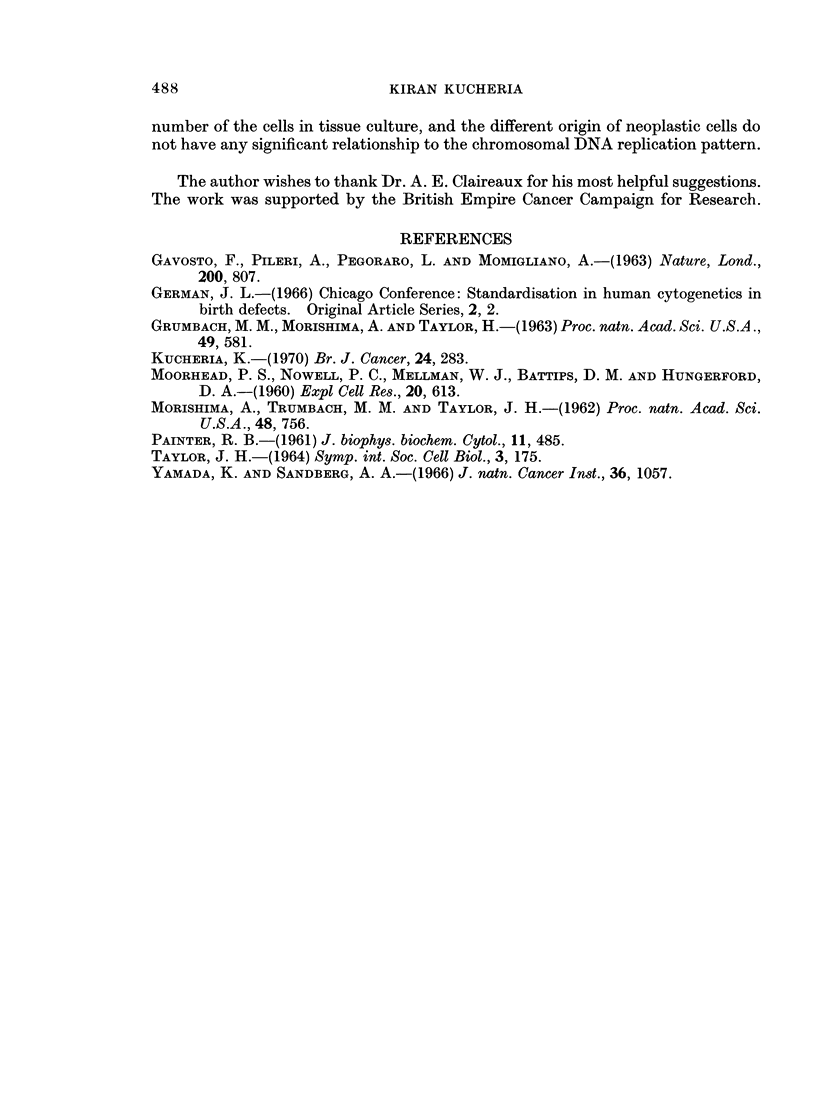# Chromosomal DNA Replication Pattern in Human Tumour Cells in vitro

**DOI:** 10.1038/bjc.1970.58

**Published:** 1970-09

**Authors:** Kiran Kucheria

## Abstract

**Images:**


### CHROMOSOMAL DNA REPLICATION PATTERN IN HUMAN TUMOUR CELLS *IN VITRO*

KIRAN KUCHERIA*

*From the Department of Morbid Anatomy, Institute of Child Health, London, W.C.*1

Received for publication March 23, 1970

**SUMMARY.—The present paper deals with the chromosomal DNA replication pattern in human solid tumour cells *in vitro*. This was studied at the terminal stages of the S-period. All the cell lines of female origin showed a late replicating chromosome in group XX6–12. In cell lines of male origin one of the chromosomes of group 21–22Y was later replicating than the rest of the members of the group. The DNA replication pattern of the autosomes and the sex chromosomes was similar to that of the cultured human leucocytes. The results of the present study show that the DNA replication pattern of the chromosome in neoplastic cells is basically unchanged despite the changes in the chromosome number and morphology. Therefore the abnormal behaviour of the neoplastic cells cannot be related to the changes in the pattern of the chromosomal DNA replication.**

AN important event in the life of a cell is mitosis, during which chromosomes are visible and cell division occurs. It has also been established that the long interval between two successive mitoses called " interphase ", includes a series of metabolic events which are important for the process of cell division. One of the metabolic events occurring during interphase is the synthesis of DNA.

The DNA synthesis pattern within an individual chromosome and among chromosomes within a cell differs a great deal. This differential pattern of the DNA replication in the chromosome is considered to be the expression of different genetic activity. In 1964, Taylor suggested, from various types of investigation on different organisms, that the morphological changes in the chromosomes, correlated with developmental stages, are indications of gene action rather than special types of DNA. The application of these aspects and techniques to the study of tumour chromosomes may be of direct relevance to the role of chromosomal DNA synthesis in cancer. Bearing this fact in mind, the present study was undertaken to examine the chromosomal DNA replication pattern in human tumour cells *in vitro* labelled with tritiated thymidine ($H^3TDR$).

### MATERIALS AND METHODS

The five cell lines used are shown in Table I which also shows their sex and the passage number at the time of investigation. At the same time, the duration of the cell cycle was also studied (for details see Kucheria, 1970).

* Present address: Department of Anatomy, All-India Institute of Medical Sciences, New Delhi-16 (India).

TABLE I

| Cell lines used | Sex | Passage number at time of investigation |
|---|---|---|
| Rhabdomyosarcoma | | |
| Rh-1 | F | 36 |
| Rh-2 | M | 9 |
| Astrocytoma | | |
| As-1 | F | 11 |
| Neuroblastoma | | |
| Ne-1 | F | 11 |
| Ne-2 | M | 8 |

Tumour samples were cultured using Eagle's medium supplemented with 10% of calf serum and grown as a monolayer. Cells from all the five cell lines were continuously labelled with $H^3$TDR, 1 $\mu$Ci/ml. (sp. activity 3 Ci/mM). Cell samples were fixed for chromosome preparations at an interval of two hours.

The chromosome preparations were made by using the modification of the technique described by Moorhead *et al.* (1960) and were stained overnight with 2% lectoacetic orcein. For autoradiography, preparations were covered with Kodak A.R. 10 stripping film; exposed for 2 weeks and finally developed in Kodak developer D 19b (Kucheria, 1970).

The chromosomal DNA replication pattern of the cells, which were labelled at the interphase by the addition of $H^3$TDR, was studied at the first mitosis after labelling. The regions of the chromosomes which replicate in the presence of $H^3$TDR were detected as labelled regions in autoradiographs. The first labelled cell to pass through division was the one at the end of the S-period during exposure to $H^3$TDR and those which pass through mitosis later were labelled in the early S-period. This makes it possible to analyse the replicatory behaviour of each chromosome. In practice the replication of chromosomes at the end of the S-period can be studied best by the continuous labelling method.

### RESULTS

The cell line Rh-1 showed 56–57, cell line Rh-2 showed 73–75 and cell lines Ne-1, Ne-2 and As-1 showed 46 as the modal chromosome numbers. In cell line Rh-1 and Rh-2, great karyotypic diversity was observed and neither of the two karyotypes showed similar distribution of chromosomes. Marker chromosomes like abnormal long acrocentrics in the cell line Rh-1 and a tiny metacentric in the cell line Rh-2 and abnormal dicentrics in both the cell lines were frequently observed. Cells from cell lines Ne-1, Ne-2 and As-1 showed apparently normal karyotypes. Details about the case histories and chromosome pattern of the cell lines are published elsewhere (Kucheria, 1970).

No labelled mitoses were observed after 2 or 4 hours continuous exposure. In cell lines of female origin (Rh-1, Ne-1 and As-1) cells fixed after 6, 8 and 10 hours of continuous labelling showed a very heavily labelled median size chromosome of the group XX6-12 (presumably the " hot X ", Fig. 2). While the rest of the chromosomes of group XX6-12 were lightly labelled or unlabelled. In cell line Rh-1 some of the cells showed two heavily labelled chromosomes (Fig. 3). In cell lines of male origin (Rh-2 and Ne-2), cells fixed at 6 and 8 hours after continuous labelling showed one of the chromosomes of group 21–22Y, more heavily labelled

(presumably the Y chromosome) than the rest of the members of the group (Fig. 4, 5).

In all the five cell lines one of the chromosome pair (presumably No. 15) of group 13–15, pair No. 17 and chromosomes of the groups 19–20 and 21–22 showed early termination of their DNA synthesis. One of the chromosome pairs of group 19–20 and 21–22 completed their synthesis later (Fig. 2, 5). Chromosome pair No. 1 did not show a consistent labelling pattern, but in most of the cells, some of the arm segments were late replicating than the centromeric region. The centromeric region of the chromosome pair No. 3 and the short arms of chromosome pairs of group 4–5 showed late replication. Two of the chromosomes of group 13–15 (presumably No. 13) showed late labelling of the long arms and the other two (presumably No. 14) showed late labelling of the centromeric region. The chromosomes No. 16 and 18 were seen synthesising very late in the S-period (Fig. 2, 5).

Marker chromosomes present in the cell lines Rh-1 and Rh-2 showed labelling pattern similar to the respective autosomes (Fig. 3).

### DISCUSSION

The chromosomal DNA replication patterns reported in the present paper were mainly studied at the end of the S-period. Due to the technical problems, it is difficult to study at the beginning of the S-period. The main limitation is the variability in the rate at which individual cells in a single population progress through the mitotic cycle. Cells labelled at a given interval in the S-period pass through mitotis during an interval of time; this interval will be greater for cells labelled earlier in the S-period.

Replication appears to be organised in terms of the function of chromosome regions during interphase. Euchromatin is genetically active, starting replication first and the inactive heterochromatic region replicates last; there is however an extensive overlap in the S-period when both types replicate simultaneously. It is a generally accepted view that the heteropyknotic X-chromosome represents the

---

### EXPLANATION OF PLATES

FIG. 1.—Karyotypes of a cell before and after preparing the autoradiograph, with 55 chromosomes from cell line Rh-1; after 10 hours of $H^3$TDR incorporation. Note the presence of one heavily labelled chromosome in group XX6-12.

FIG. 2.—Karyotypes of a cell before and after preparing autoradiograph, with 46 chromosomes from cell line As-1; after 6 hours of $H^3$TDR incorporation. Note the presence of one heavily labelled chromosome in group XX6-12; chromosomes No. 15, 17 and one of the pairs in groups 19–20 and 21–22 unlabelled.

FIG. 3.—Karyotypes of a cell before and after preparing autoradiograph, with 57 chromosomes from cell line Rh-1; after 10 hours of $H^3$TDR incorporation. Note the presence of two, very heavily labelled chromosomes in group XX6-12.

FIG. 4.—Karyotypes of a cell before and after preparing autoradiograph, with 66 chromosomes from cell line Rh-2; after 12 hours of $H^3$TDR incorporation. Note the presence of one densely labelled chromosome in group 21–22Y than the rest of the members of the group.

FIG. 5.—Karyotypes of a cell before and after preparing autoradiograph, with 46 chromosomes from cell line Ne-2; after 10 hours of $H^3$TDR incorporation. Note the presence of one densely labelled chromosome in group 21–22Y than the rest of the members of the group; unlabelled No. 17 and one of pairs of group 19–20 and 21–22.

FIG. 6.—Metaphase spreads before and after preparing autoradiograph, with 109 chromosomes from cell line Rh-1. Arrows indicate the peripheral position of the two late labelling chromosomes.

1

Kucheria

2

Kucheria

3

Kucheria

Kucheria

Kucheria

6

Kucheria late labelling X-chromosome observed in autoradiographic studies and the Barr body in resting nuclei.

In cell line Rh-1, one to two late labelling chromosomes in group XX6-12 were observed. In cells with 108 chromosomes the presence of the two late labelling chromosomes may be due to the tetraploid complement of the X-chromosome and extra chromosomes may be due to additional autosomes. In cells with 56 to 58 chromosomes, one late labelling chromosome might represent the diploid set of the X-chromosomes. However, some cells (from Rh-1) with 57 and 107 chromosomes had two and some with 55, 107 and 110 chromosomes had only one heavily labelled chromosome present among the members of XX6-12 group.

It is to be seen whether the heavily labelled chromosome observed under the conditions of the present study of tumour cells *in vitro* is the same as the heavily labelled chromosome of the normal cell. Identification of the X-chromosome on morphological ground only, especially in tumour cells, is very difficult. It has been suggested (Grumbach *et al.*, 1963; Morishina *et al.*, 1962) that in flattened preparations of metaphase cells, the late labelling X-chromosome is positioned at the pheriphery more frequently than would be expected by chance distribution of the chromosomes. A similar peripheral position of the late labelling chromosome was observed in the cells from cell lines Rh-1, As-1 and Ne-2. The other support for the identity of one of the X-chromosome is on the pattern of its DNA labelling and it is thought to be the last in group XX6–12 to terminate its DNA synthesis. A late labelling X-chromosome is not found in the normal male (XY) cells where as it is present in normal female (XX) cells.

The morphology, size, position and the DNA replication patterns of the late labelling chromosome observed in the cell from cell lines Rh-1, As-1 and Ne-2 are similar to that of the late replicating X-chromosome observed in cultured blood cells of normal human females. Their variation in number, in different cells (Rh-1) could be attributed either to the conditions effecting the long term tissue culture or to the process which was involved in their origin.

The present studies of the five solid tumours *in vitro* showed that the chromosome DNA replication pattern in cells containing either more or less chromosomes than the modal cells was similar to that of the modal cells. In fact the DNA replication pattern of the chromosomes in the modal cells (tumour cells) is similar to that of the cultured normal human leucocytes (German, 1966), especially in respect to the early termination of the DNA synthesis of the chromosomes number 17 and 19–20. Late DNA replication of one of the X-chromosomes in the female cells has also been reported in other neoplastic cells by some authors. Painter (1961) observed them in HeLa S3 cells, Gavosto *et al.* (1963) in leukaemic aneuploid leucocytes and Yamada and Sandberg (1966) in cancer effusions cultured for short periods. The last authors found similarities to the normal replication pattern of some autosomes.

The results of the present studies show clearly that the DNA replication pattern of the chromosomes in neoplastic cells is basically unchanged despite changes in chromosome number and morphology (ref. to the cell lines Rh-1 and Rh-2 with chromosome modes at 56–57 and 73–75 respectively and presence of marker chromosomes). This indicates that the abnormal behaviour of the neoplastic cells cannot be related to the changes in the pattern of the chromosomal DNA replication. The present results also show that the age of the patient, the passage number of the cells in tissue culture, and the different origin of neoplastic cells do not have any significant relationship to the chromosomal DNA replication pattern.

The author wishes to thank Dr. A. E. Claireaux for his most helpful suggestions. The work was supported by the British Empire Cancer Campaign for Research.

### REFERENCES

GAVOSTO, F., PILERI, A., PEGORARO, L. AND MOMIGLIANO, A.—(1963) *Nature, Lond.*, **200**, 807.

GERMAN, J. L.—(1966) Chicago Conference: Standardisation in human cytogenetics in birth defects. Original Article Series, **2**, 2.

GRUMBACH, M. M., MORISHIMA, A. AND TAYLOR, H.—(1963) *Proc. natn. Acad. Sci. U.S.A.*, **49**, 581.

KUCHERIA, K.—(1970) *Br. J. Cancer*, **24**, 283.

MOORHEAD, P. S., NOWELL, P. C., MELLMAN, W. J., BATTIPS, D. M. AND HUNGERFORD, D. A.—(1960) *Expl Cell Res.*, **20**, 613.

MORISHIMA, A., TRUMBACH, M. M. AND TAYLOR, J. H.—(1962) *Proc. natn. Acad. Sci. U.S.A.*, **48**, 756.

PAINTER, R. B.—(1961) *J. biophys. biochem. Cytol.*, **11**, 485.

TAYLOR, J. H.—(1964) *Symp. int. Soc. Cell Biol.*, **3**, 175.

YAMADA, K. AND SANDBERG, A. A.—(1966) *J. natn. Cancer Inst.*, **36**, 1057.